# Usefulness of electrohydraulic lithotripsy gel-immersion cholangioscopy for giant common bile duct stones

**DOI:** 10.1055/a-2784-8242

**Published:** 2026-02-13

**Authors:** Hiroki Yamana, Hideki Kamada, Kiyoyuki Kobayashi, Naoki Fujita, Daisuke Namima, Hideki Kobara

**Affiliations:** 1 12850 Department of Gastroenterology and Neurology, Faculty of Medicine, Kagawa University, Kagawa, Japan

Electrohydraulic lithotripsy (EHL) is an effective treatment for large common bile duct (CBD) stones. However, endoscopic visualization can be impaired by turbid bile or stone fragments/debris generated during the procedure. Gel-immersion endoscopy using a transparent, viscous gel (Viscoclear; Otsuka Pharmaceutical Factory, Tokushima, Japan 1 ) has been reported as an effective method for securing the visual field 2 3 . We employed this technique to secure the cholangioscopic visual field during EHL.

**Fig. 1:**
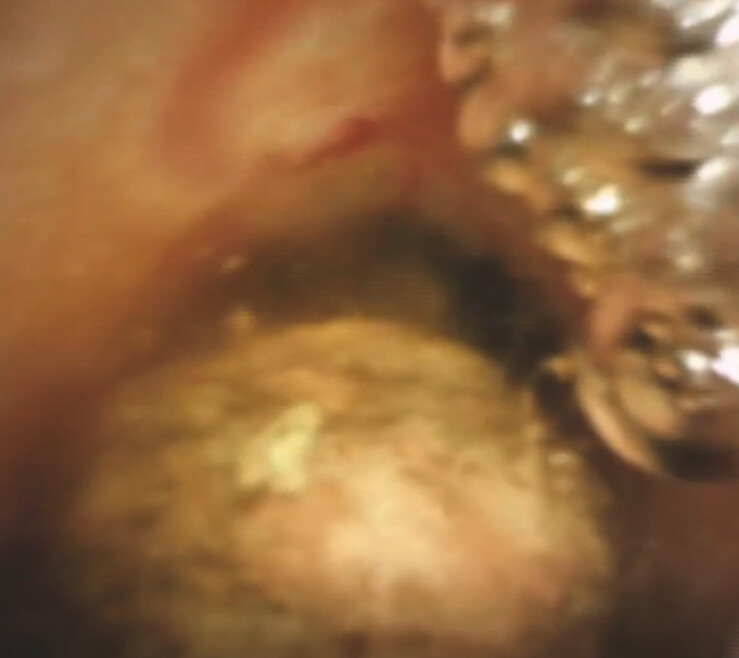
We observed common bile duct stones using a digital cholangioscope.

A 74-year-old woman was admitted to our hospital for the treatment of a large CBD stone. EHL was performed after endoscopic retrograde cholangiopancreatography was attempted. A digital cholangioscope (SpyGlass DS; Boston Scientific, Marlborough, MA, USA) via a 0.025-inch guidewire was inserted into the CBD. A CBD stone (diameter: 20 mm) was successfully observed ( Fig. 1 ).

We performed EHL using an EHL probe combined with an electrohydraulic shockwave generator (Autolith Touch; Boston Scientific, Marlborough, MA, USA 4 ). The EHL generator was set to high power at a rate of 30 pulses/s. Although normal saline is generally used for CBD filling during EHL, it induces poor visual fields. To overcome this problem, ViscoClear was used ( Video 1 ).

Gel-immersion electrohydraulic lithotripsy was performed to treat a large common bile duct stone.Video 1

**Fig. 2:**
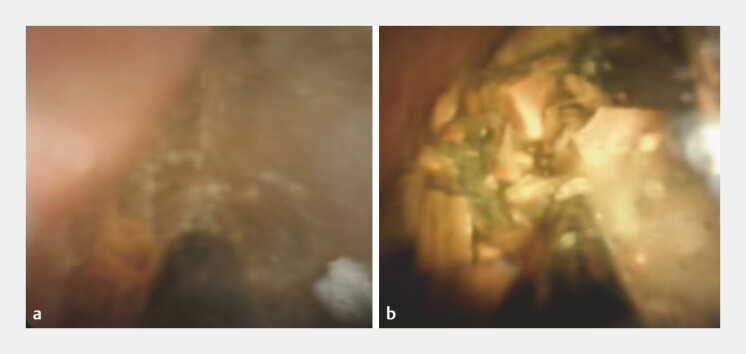
**a** When using saline solution, our visibility was impaired by fragments of scattered stones and debris. **b** Compared to the use of saline solution, we found it easier to see clearly when using the gelatinous liquid.

**Fig. 3:**
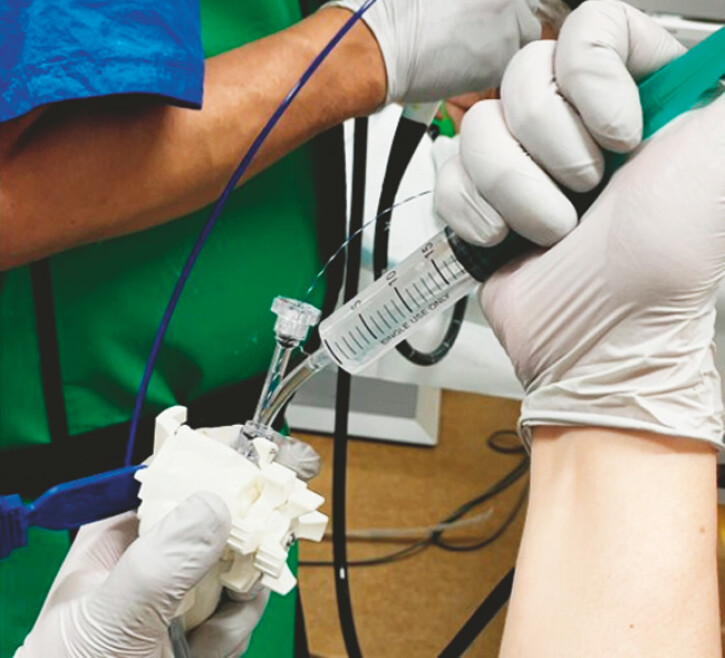
We slowly inject the gel into the forceps channel port using a 10 ml or 20 ml syringe, adding 1–2 ml at a time.

During EHL, the maintained visual field was clearer using Viscoclea than using saline irrigation, even in the presence of scattered stone fragments and debris ( Fig. 2 ). When visualization deteriorated during the procedure, injecting 1–2 mL of the gel restored the field of view ( Fig. 3 5 ). A total of 30 mL of gel was used for this procedure. The procedure was safely completed while maintaining a clear visual field. The stones were successfully fragmented using approximately 1,000 shockwaves under a clear visual field. In conclusion, this procedure effectively improves endoscopic visibility and may reduce the risk of increased bile duct pressure when a small amount of gel is used.

Endoscopy_UCTN_Code_TTT_1AR_2AH
